# Effectiveness of a minimal psychological intervention to reduce mild to moderate depression and chronic fatigue in a working population: the design of a randomized controlled trial

**DOI:** 10.1186/1471-2458-13-129

**Published:** 2013-02-12

**Authors:** Ed Aelfers, Hans Bosma, Inge Houkes, Jacques ThM van Eijk

**Affiliations:** 1 Maastricht University, Research school CAPHRI, Social Medicine, P.O. Box 616, 6200 MD, Maastricht, The Netherlands; 2 ARBOdienst DSM, Occupational Health Services, P.O. Box 27, 6160 MB, Geleen, The Netherlands

## Abstract

**Background:**

In a working population, common mental complaints like depressed mood and chronic fatigue are highly prevalent and often result in further deterioration of mental health and consequently absence from work. In a large occupational health setting, we will evaluate the (cost-) effectiveness of a Minimal Psychological Intervention (MPI), in reducing symptoms of depression and chronic fatigue in a working population. The MPI is also evaluated regarding its appreciation by worker, nurse, and occupational health physician (process evaluation). The tailor-made intervention is administered by nurses, who are trained in the principles of cognitive behavioural therapy and self-management.

**Methods/design:**

The presented WoPaCoM study (Work Participation of Workers with Common Mental complaints) is a two-armed randomized controlled trial, comparing MPI with usual care. A total number of 124 workers suffering from (chronic) mental fatigue or mild to moderate depression will be included. A stratified and block randomization will be applied, stratifying by customer organisation, income, and gender, using a block size of four. It will include a baseline measurement and subsequently follow up measurements after 4, 6 and 12 months. The primary outcome measures are symptoms of either fatigue (using the Checklist Individual Strength) and/or depression (using the Beck Depression Inventory) and secondary outcome measures include sickness absence, self efficacy, costs and quality of life. Analysis will include both univariate and multivariate techniques and data will be analysed according to the intention to treat principle.

**Discussion:**

Patient recruitment in an occupational setting proves to be complicated and time consuming. Shift work for instance proved to be an obstacle for making appointments for consultation with the nurse. Furthermore, economic developments might have created job insecurity which negatively influenced participation in the study, with workers being anxious to be detected as having psychological problems. Additionally, long-term follow-up in a working population is time-consuming and continuously engages occupational health staff and administrative personnel to control the process of data gathering. However, if the intervention proves to be effective, occupational medicine will have a manageable option for treatment of workers who are at risk of loss of productivity or sickness absence.

**Trial registration:**

Nederlands Trialregister NTR3162

## Background

Fatigue and psychological distress are fairly common symptoms in the working population [1-3]. Approximately 20% of the working population report symptoms that fit the concept of prolonged fatigue [4]. There is evidence that fatigue is correlated with lost productive work time and related costs [5,6]. Fatigue is a predictor of sickness absence [6], future disability pension [7], and even occupational accidents [8]. There is also evidence that workers visiting their GP with complaints of fatigue often have higher levels of depressive complaints [9-11]. Problematic is that such mental health complaints often go unrecognized in practice [4]. By ignoring these complaints, symptoms may increase and eventually result in sickness absence and work-related disability [12-14]. From a preventive point of view, it is essential not to wait until workers are reported ill. It is important to intervene at an early stage to obviate aggravation of complaints.

In cases of minor depression, drug-related therapy is not necessarily the first choice [15-17]. A number of evidence-based psychological interventions, such as cognitive behavioral therapy and self-management strategies, are preferable for such complaints. Based on these principles, we previously developed a Minimal Psychological Intervention, which – in order to decrease the physicians’ work pressure – can likely be carried out by specially-trained nurses [18-20]. In the DELTA Study (Depression in Elderly with Long-Term Afflictions), it was found that the MPI provided an additional professional tool for nurses who cared for the chronically ill elderly persons with mild to moderate depressive symptoms [21]. The MPI was found both acceptable and feasible. The intervention improved quality of life and reduced the depressive symptoms [22,23].

In the current study, we set out to examine whether the MPI – as delivered by occupational nurses – is equally effective in a working population aimed at workers’ complaints of both depressive symptoms and fatigue. In this WoPaCoM study (Work Participation of workers with Common Mental complaints) we had the following main research questions:

1. What is the effect of the occupational nurse-delivered MPI on the mental health status, quality of life, and labor participation of workers with (symptoms of) mental fatigue and/or mild to moderate depressive symptoms, in comparison with care as usual?

2. How do workers, occupational nurses, and occupational physicians appreciate the intervention? What possible barriers regarding implementation do they experience?

## Design and methods

### Design

**Figure 1:**
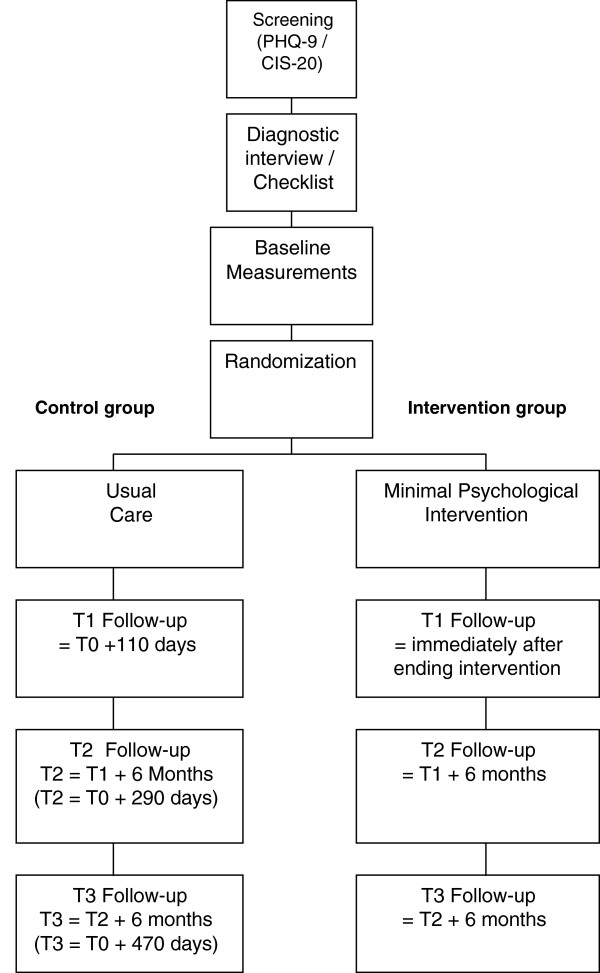
Flow-chart WoPaCom study.

WoPaCoM is a study conducted in an Occupational Health Service in the South of The Netherlands that started at the end of 2007. Data gathering was finished in 2012. The customers of this service have their activities in the chemical industry. The two main customer organizations consist of approximately 6,000 workers in several settings, jobs and positions, varying from white and blue collar workers to researchers and administrative personnel. The WoPaCoM study is a two-armed (stratified) randomised controlled trial (with a block size of 4), evaluating the effectiveness of the MPI, including a process evaluation, for decreasing symptoms of fatigue and depression. After the baseline measurement, follow-up measurements at 4, 6 and 12 months are carried out (Figure 1). A total number of 124 participants will be included. The effect of MPI will be compared with care as usual. The ethics committee of the Maastricht University Medical Center found that the study was not subject to the Dutch Medical Research Involving Human Subjects Act (WMO): subjects were not subjected to invasive treatments, the subjects’ privacy was sufficiently guaranteed, and the setting was one in which regular occupational care was evaluated.

### Setting and recruitment

The study takes place at an occupational health service which performs periodical preventive medical check-ups once every four years. For the current study, two questionnaires were added in order to screen for symptoms of chronic fatigue and symptoms of depression.

The CIS-20 (Checklist Individual Strength) is a 20-item self-administered questionnaire and measures several aspects of fatigue being subjective feeling of fatigue, concentration, motivation, and physical activity [24,25]. The items are scored on 7-point Likert scales (1 = “Yes, that is true”, to 7 = “No, that is not true”). Higher scores indicate a higher degree of fatigue, more concentration problems, reduced motivation, and less activity. A cut-off point of >76 was used to define fatigue in a working population [26].

The PHQ-9 (Patients Health Questionnaire) consists of nine questions regarding the prevalence and intensity of symptoms of depression over the last two weeks. The response options are: “Not at all”, ”Several days”, “More than half the days” and “Nearly every day”. Its brevity and the fact that it is a self-administered questionnaire make it a useful tool in screening for depression in primary care. The PHQ-9 has been validated for both diagnosing depression and measuring severity [27-29] and even for evaluating treatment effects [30,31]. All participants who scored at least two depressive symptoms at least at “more than half the days” and at least one of these symptoms was depressed mood or anhedonia, were eligible. Depressed mood was present with a PHQ score of >4 (mild depression), but <20 (moderately severe depression) [32].

Workers with heightened scores of either fatigue and/or depressed mood were invited for an additional diagnostic interview to confirm their eligibility and to check for exclusion criteria. The Mini International Neuropsychiatric Interview (MINI) was used to confirm the diagnosis from the PHQ-9. The interview was administered by a trained nurse. The MINI is a validated and reliable diagnostic structured interview covering 17 disorders based on DSM-IV criteria [33,34]. A visual analogue scale was also used to determine the negative impact of the complaints on daily life functioning (ranging from 0 (no impact) to 100 (extreme impact)). A cut-off point of 30 was arbitrarily chosen. Participants were excluded if the MINI indicated a major depression in combination with a PHQ-9 score of 14 or higher, in case of recent (medical) events as an explanation for fatigue, serious psycho-social problems, former episodes of depression or bipolar disorder or psychiatric co-morbidity, passive death wish or suicidal thoughts, and alcohol or drug dependency (Table 1). If participants met the criteria for fatigue and depressed mood, exceeded the score of 30 on the impact visual analogue scale they were asked for informed consent. Serious psychiatric conditions would have been referred further (e.g. to physician), but this did not occur in our study.

**Table 1:** Inclusion and exclusion criteria as applied by the occupational nurse

**Inclusion criteria:**	**Exclusion criteria:**
- Score PHQ-9 > 3 < 14	- Severe / major depression
- Score CIS-20 > 76	- Major psychiatric problems ( e.g. bipolar depression, schizophrenia, suicidal risk)
- Burden in daily life > 30 (visual analogue 0–100)	- Current psychological/psychiatric treatment
	- Relevant clinical disease
	- (Drug) Addiction(s)
	- Recent (life) events strongly related to fatigue and depression (e.g. recent (major) surgery)

### Randomisation

After having completed the baseline questionnaire, participants were randomly assigned to either the intervention or control group to ensure equal numbers in both groups. Furthermore, the randomisation was stratified to avoid possible confounding. The strata are the customer organization (representing different production plants and organisational structure), income (low, high as defined by the median income in the organization), and gender (male, female). The intervention group received the Minimal Psychological Intervention. The control group received care as usual, being the standard follow-up by the occupational health physician. Mostly this consists of additional consultation and follow-up advice. The principles underlying the intervention (cognitive behavioural therapy and self-management) have been validated in previous research [35,36] and we therefore think that our findings are not substantially biased by the Hawthorne effect. All possible co-interventions were registered in both intervention as well as control group.

### Minimal Psychological Intervention

The intervention was carried out by a trained occupational nurse. During a period of at most four months, participants assigned to the study group had a minimum of 1 and a maximum of 10 consultations with the nurse. All consultations took place on an individual basis. There were no group sessions. The number of and time between visits depended on the participant’s progress and was thus tailor-made. The Minimal Psychological Intervention contains elements from the Chronic Disease Self-Management Program (CDSMP) by Lorig and Gonzales [37], the Reattribution model from Goldberg [38] and from the work of the project group of the Interventie Studie Eerste Lijn (INSTEL) [39]. The intervention aims at teaching workers to take responsibility for day-to-day management of their problems and its consequences for daily functioning.

The MPI consists of five phases: Phase 1: The nurse explores the participants’ cognitions on the origin of symptoms and complaints, and their relation to limitations and behaviour. Phase 2: The participant keeps a diary, where he or she records symptoms, complaints, thoughts, worries, related feelings, and behaviour. Phase 3: Using information from the diary, the nurse challenges the participant to link his behavioural strategies to his or her complaints. Phase 4: Introduction of the self-management approach by the nurse. The participant explores his or her possibilities to alter his or her behaviour. He or she then makes a plan on how to solve perceived problems and sets specific goals to be reached before the next consultation with the nurse. Phase 5: Evaluation of the progress in achieving the goals.

After a participant has completed these five phases successfully, he or she is supposed to be able to apply the self-management approach to any situation or problem he or she may encounter in the future. Nurses performing the diagnostic interview with a particular participant did not perform the MPI in that particular participant.

### The training program for nurses

During four 8h sessions, with 2-week time intervals, and the last with a four-week-interval, five nurses were trained by experienced trainers (a behavioural scientist with experience in cognitive behaviour therapy, a psychiatrist and a pharmacist) on how to apply both the diagnostics as well as the intervention. In between training days, nurses practised their newly learned skills on a pilot patient. At the end of training, two of the nurses who showed the most affinity with and best availability for performing the MPI were selected to participate in the intervention. As mentioned earlier, the training program has previously shown to be feasible, attractive and successful among nurses [18-20], but is evaluated again in the current study. Booster sessions were held monthly during the first part of the study and then gradually scaled down depending on the specific needs of the nurses; both a psychiatrist and a psychologist could be contacted by telephone to discuss cases at any time.

### Data collection

Data are collected at four points in time: at baseline (T0), one week after the intervention period (T1), and at six and twelve months after the intervention period (T2, T3) (Figure 1). The intervention period for participants allocated to the intervention group varies from one week to four months. T1 (the first follow-up phase) for the control group was set at 4 months, which was the estimated mean duration of the intervention period in the intervention group. Data were collected using self-administered questionnaires (Figure 1). Workers and nurses allocated to the intervention group were well aware of the allocated arm. Randomisation and data collection was carried out by separate administrative personnel members without any competing interests.

### Effect evaluation

Table 2 provides an overview of the outcome measures. The primary outcome measures in this study are fatigue as measured with the CIS-20 and the level of depression as measured with the Beck Depression Inventory (BDI) [40,41]. Secondary outcome measures in the study are quality of life as measured with the SF-36 [42], Mastery using the Personal Mastery Scale developed by Pearlin and Schooler [43], and General Self Efficacy [44,45]. Sick-leave data were available from official plant registrations. Varying covariates were measured. Information on demographic factors (age, gender, marital status, religion, education, occupation, and income) was collected in the screening phase. Other measures were coping using the active coping, avoidant coping and passive coping scales from the Utrecht Coping List (UCL) [46], anxiety assessed using the anxiety subscale from the Symptom Checklist (SCL-90) [47], co-morbidity using a 24-item chronic conditions list (e.g. heart disease, cancer) and 16-item life-events list where patients report which life events they have experienced in the past year, and how they value these events (positive, negative, or neutral). To check for contamination in the control group, two questions are added in the T3 questionnaire asking whether or not workers in the control group had heard, used parts of, or somehow benefited from the MPI.

**Table 2:** List of instruments (questionnaires/variables)

**Variable/ used instrument**	**Moment in time**				
	PMCU*	**T0**	**T1**	**T2**	**T3**
Organization	X				
Income	X				
Gender	X				
Age	X				
PHQ-9 [27-29]	X	X			
CIS20 [25,26]	X	X	X	X	X
SF-36 [40]		X	X	X	X
BDI [38]		X	X	X	X
SCL (Anxiety scale) [40]		X	X	X	X
UCL [44]		X	X	X	X
Pearlin & Schooler Mastery [41]		X	X	X	X
Sherer/Bosker (GSE) [42]		X	X	X	X
Prodisq (module C-E) [46,47]		X	X	X	X
Chronic disease		X			X

*PMCU Periodic Medical Check Up.

### Economic evaluation

For an economic evaluation, several modules of the PROductivity and DISease Questionnaire (PRODISQ) were used for the measurement of productivity costs. The modular questionnaire covers all relevant aspects of the relationship between health and productivity. In this study we will concentrate on productivity of individual participants. We used several PRODISQ modules including absence from work (module C), compensation mechanisms that may reduce productivity loss (module D), and reduced productivity at work (module E) [48,49]. Absence from work is measured as the total number of lost working days as well as the number of sick-leave episodes within a period of three months. Compensation mechanisms include the compensation of sick leave days by either the worker himself or a colleague, with or without extra productivity cost. Productivity cost being differentiated in the amount of work and the quality of work.

### Analysis

Data will be analysed according to the intention to treat principle. Despite participants dropping out of the intervention or not returning follow-up questionnaires, each individual is analysed as randomized (either control or intervention). We tried to encourage (potential) drop-outs to return their questionnaires in order to have complete data as much as possible. But if this was not possible, available (but not complete) data will still be included in the multilevel repeated measures analysis; this type of analysis better allows incomplete data. Finally, in the forthcoming articles, we will give full insight into the response rates at all phases of the study and consideration of the extent to which this might have caused specific biases.

Changes in primary and secondary outcome measures between the intervention and the control group will be analysed using both univariate and multivariate techniques. Models will be adjusted for baseline differences, age, gender, and socioeconomic status. Potential additional confounding factors and effect modifiers (covariates) will be checked separately and when necessary included in the model. If numbers permit, subgroup analyses will study the robustness of the findings across organizations and workers with a low and high income.

### Power calculation

Using an individual randomization scheme, assuming an α of 0.05, a 1 – β of 0.90, and a decrease in symptoms of depression and/or fatigue of 25 percent in the intervention group versus a 5 percent decrease in the control group, 62 persons per arm were needed. As we expected that 25% percent would stop participating after baseline (attrition), 83 persons per arm had to be initially recruited [50,51].

### Process evaluation

The aim of the process evaluation is to assess how the intervention is perceived e.g. appreciated by all participants (i.e. workers and nurses). Using the framework of Jonkers and colleagues [19], it focuses on the following outcomes. The reach of the intervention, being defined as the proportion of the intended target population that actually participated in the intervention. The dose delivered, defined as the completeness of the intervention and number and duration of the intervention visits. Dose received, is described in two concepts, namely exposure and satisfaction. Exposure is the extent to which participants actively engage with and are receptive to the intervention, and satisfaction is defined as participants's satisfaction with the intervention [52]. Barriers are described as the extent to which problems were encountered during the intervention. Data were collected using questionnaires filled out by nurses after every intervention visit, by means of checklists that were kept by the nurse for every participant (regarding which steps of the intervention had been taken), and by questionnaires filled out by participants after the end of the intervention.

## Discussion

### Progress of the study

In 2007, based on the occupational health service’s experience, it was estimated that it would take approximately two years to recruit participants. However, both the participation rates to the medical check-ups and the return rates of the screening questionnaires were smaller than expected and decreased substantially, particularly during the first phase of the economic crisis in Europe (2009). Increased perceptions of job insecurity might underlie these trends. Increasing efforts were needed to keep participants involved in the intervention itself, but also during the one-year follow-up. We ended inclusion in the study in April 2011 and the intervention has been administered to all participants in the intervention arm. Data collection was completed in June 2012. 127 Participants have completed their follow-up questionnaires. Of all participants initially included in the study 93% were male and 7% female (which was expected given the gender distribution in the plants). As per the stratified and blocked design, there is an equal number of intervention and control participants in the different strata.

### Future implementation

If this intervention proves to be effective in reducing depression and fatigue and in improving quality of life, implementation of the intervention in the occupational health care system is considered and anticipated. An implementation and dissemination plan will be developed that might be of use for dissemination beyond the study plant. Additional arguments for such dissemination and implementation could be derived from positive changes in sick leave data and productivity figures.

## Competing interests

The author(s) declare that they have no competing interests.

## Authors’ contributions

EA is investigator and wrote the manuscript, with input from the other authors. IH is investigator, HB is supervising the planning and progress of the project, and JvE is the principal investigator and author of the study protocol. All authors read, edited, and approved the final manuscript.

## Pre-publication history

The pre-publication history for this paper can be accessed here:

http://www.biomedcentral.com/1471-2458/13/129/prepub
